# Identifying Good Responders to Glucose Lowering Therapy in Type 2 Diabetes: Implications for Stratified Medicine

**DOI:** 10.1371/journal.pone.0111235

**Published:** 2014-10-23

**Authors:** Angus G. Jones, Beverley M. Shields, Christopher J. Hyde, William E. Henley, Andrew T. Hattersley

**Affiliations:** 1 NIHR Exeter Clinical Research Facility, University of Exeter Medical School, Exeter, United Kingdom; 2 Public Health and Epidemiology, University of Exeter Medical School, Exeter, United Kingdom; 3 Institute of Health Research, University of Exeter Medical School, Exeter, United Kingdom; University of Milan, Italy

## Abstract

**Aims:**

Defining responders to glucose lowering therapy can be important for both clinical care and for the development of a stratified approach to diabetes management. Response is commonly defined by either HbA1c change after treatment or whether a target HbA1c is achieved. We aimed to determine the extent to which the individuals identified as responders and non-responders to glucose lowering therapy, and their characteristics, depend on the response definition chosen.

**Methods:**

We prospectively studied 230 participants commencing GLP-1 agonist therapy. We assessed participant characteristics at baseline and repeated HbA1c after 3 months treatment. We defined responders (best quartile of response) based on HbA1c change or HbA1c achieved. We assessed the extent to which these methods identified the same individuals and how this affected the baseline characteristics associated with treatment response.

**Results:**

Different definitions of response identified different participants. Only 39% of responders by one definition were also good responders by the other. Characteristics associated with good response depend on the response definition chosen: good response by HbA1c achieved was associated with low baseline HbA1c (p<0.001), high C-peptide (p<0.001) and shorter diabetes duration (p = 0.01) whereas response defined by HbA1c change was associated with high HbA1c (p<0.001) only. We describe a simple novel method of defining treatment response based on a combination of HbA1c change and HbA1c achieved that defines response groups with similar baseline glycaemia.

**Conclusions:**

The outcome of studies aiming to identify predictors of treatment response to glucose lowering therapy may depend on how response is defined. Alternative definitions of response should be considered which minimise influence of baseline glycaemia.

## Introduction

Being able to identify patients who respond particularly well or poorly to a therapy is important for both the study and application of a stratified (personalised) approach to the management of diabetes. Studies aiming to determine predictors and mechanisms of altered treatment response to a therapy may select patients with extremes of response for intensive phenotyping to maximise power (e.g. DIRECT study, www.direct-diabetes.org), or categorise response as part of their analysis. In clinical care identifying when a therapy has been ineffective and can be stopped may benefit both the patient (e.g. avoiding side effects from ineffective therapy) and care provider (reduced cost); this approach has been incorporated into clinical guidance in the UK advising discontinuation of many more expensive glucose lowering treatments if response criteria are not achieved [1].

Response to glucose lowering therapy is conventionally defined in one of two ways, the absolute change in HbA1c after treatment or whether a target HbA1c is achieved. Existing studies aiming to identify clinical and biomarker predictors of glycaemic response have not used a consistent approach. How these methods differ in both the individuals they identify and their associated characteristics is unclear and has not been previously explored.

We aimed to determine the extent to which the individuals identified as markedly good responders to glucose lowering therapy, and their characteristics, depend on the response definition chosen. We describe a simple alternative method of defining treatment response based on a combination of HbA1c change and HbA1c achieved that may have advantages over existing definitions when studying treatment response in diabetes.

## Methods

### Study population

We prospectively studied 230 non-insulin treated participants with HbA1c ≥58 mmol/mol (7.5%) commencing GLP-1A therapy as part of their usual diabetes care recruited to Predicting Response to Incretin Based Agents (PRIBA) study (http://clinicaltrials.gov/show/NCT01503112). We measured HbA1c at baseline and 3 months (10–14 weeks) and assessed the following baseline clinical characteristics: HbA1c, BMI, duration of diabetes, age of diabetes diagnosis, glucose, C-peptide, triglycerides, creatinine. Blood tests were performed fasting.

To avoid confounding by co-treatment change and adherence we limited our analysis to the 169 subjects who remained on treatment at 3 months, had no other glucose lowering treatment increased or stopped and had >80% self-reported adherence over the 2 weeks prior to HbA1c assessment.

### Conventional response definitions

To assess whether conventional definitions of response (based on absolute change in HbA1c or HbA1c achieved on therapy) identified similar individuals and associated characteristics we defined good response to GLP-1A therapy (‘responders’) as follows:

1. HbA1c change (from baseline to 3 months)<−30 mmol/mol (−2.7%) (n = 38)
2. HbA1c achieved (HbA1c at 3 months on therapy) <56 mmol/mol (7.3%) (n = 38)

Definitions were chosen based on the quartile of best responders for each method, using the closest thresholds which allowed equal numbers of responders. ‘Non responders’ were defined as those not achieving these criteria within each category. We assessed agreement in classification of responders between different response definitions by calculating percentage agreement and Kappa statistic and assessed differences in baseline characteristics using Mann-Whitney U (continuous variables) or Chi Squared (dichotomous variables).

### Novel response definitions

We developed a novel alternative method to define good responders to therapy, combining HbA1c achieved and HbA1c change using cut-offs based on the same centile so that only those meeting both criteria are designated responders. For comparison to the above methods the following definition was chosen to give 38 responders again (equating to the 41^st^ centile of response for each definition):

HbA1c change<−21 mmol/mol (−1.9%)

AND

HbA1c achieved <62 mmol/mol (7.8%)

We also defined response groups using HbA1c change, adjusted for baseline, using linear regression, with responders defined as the 38 participants with the greatest baseline adjusted HbA1c change (HbA1c change unstandardised linear regression residual ≤ −9.7 mmol/mol).

We examined the characteristics of responders and ‘non-responders’ (remaining participants) for both methods and assessed the extent to which these two methods identify the same responders and associated characteristics.

### Statistical analysis

All statistical analysis was performed using PASW Statistics for Windows, Version 18.0. Chicago: SPSS Inc.

### Laboratory analysis

HbA1c, creatinine, triglycerides and glucose were measured in recruitment centres’ local laboratories (all CPA accredited NHS blood science laboratories). HbA1c measurement was standardised to the IFCC reference method procedure, all repeated measurements within the same individual were analysed within the same laboratory. C-peptide was measured in the Biochemistry Department at the Royal Devon and Exeter Hospital, Exeter, UK, using the routine automated E170 immuno-analyser from Roche Diagnostics (Manheim, Germany).

### Ethical approval

The study was approved by the South West Research Ethics Committee (UK). Written informed consent was obtained from all participants.

## Results

### Participant baseline characteristics

Baseline characteristics of participants are shown in **Table 1**.

**Table 1:** characteristics of included participants at study baseline.

Baseline characteristic	Median (IQR)
HbA1c (mmol/mol )	85 (73–98)
HbA1c (%)	9.9 (8.8–11.1)
% male	52
Age (years)	54 (47–60)
Age at diabetes diagnosis (years)	46 (40–52)
Diabetes duration (years)	6 (3–10)
BMI (kg/m^2^)	40.0 (35.5–44.7)
Creatinine (µmol/L)	70 (56–84)

### Conventional definitions of response identify different individuals as treatment responders

**Figure 1:**
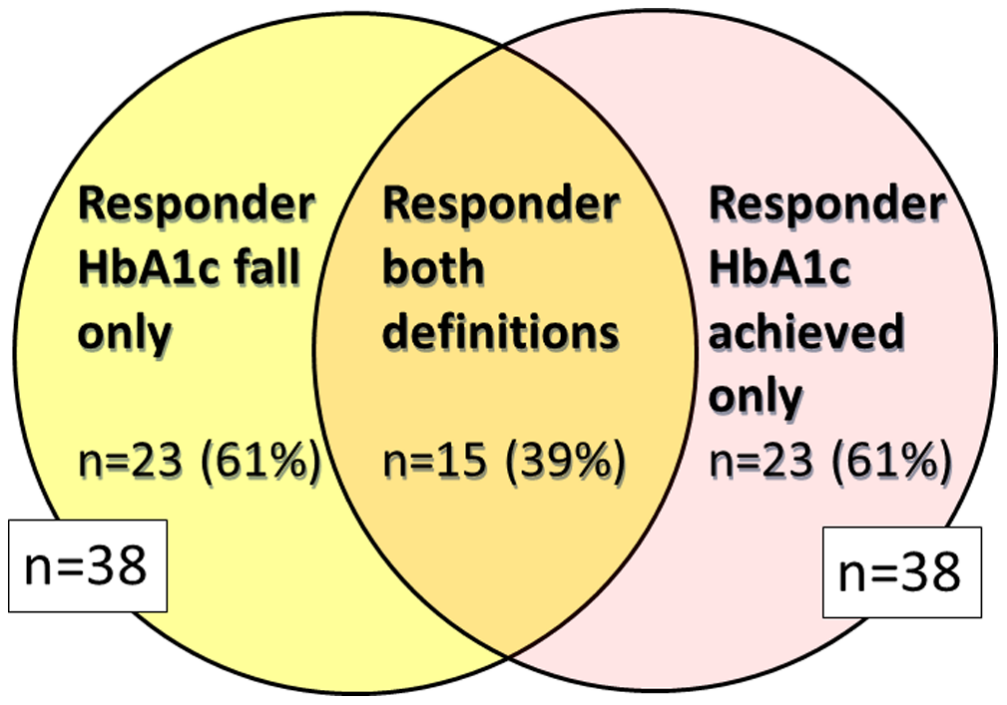
Different methods of defining good responders to glucose lowering therapy identify different individuals. Response definitions based on the top quartile of response for each method to give equal numbers of responders, n = 169.

Agreement between the two conventional definitions of response was poor (Kappa statistic 0.22; 0 = no agreement, 1 = perfect agreement [2]). Only 15 (39%) of responders by one definition (Hba1c achieved or HbA1c change) were also responders using the alternative definition of good response **(** **Figure 1** **)**.

### Those identified as good responders using different definitions have very different baseline HbA1c and associated characteristics

The characteristics of responders by both conventional definitions are shown in **Table 2**. Using a response definition based on absolute change predominantly identifies those with high baseline HbA1c whereas using a definition based on HbA1c achieved will select those with a low baseline (median baseline HbA1c 101 vs 74 mmol/mol (11.4 vs 8.9%), p<0.001). Both duration of diabetes and fasting C-peptide are significantly different in responders selected by the two definitions, these are variables that are correlated with baseline HbA1c (duration of diabetes Spearmans’s r = 0.19, p = 0.01, C-peptide r = −0.30, p = 0.001).

**Table 2:** baseline characteristics of responders to GLP-1A therapy defined by HbA1c achieved or HbA1c change.

	Responder HbA1c achieved (n = 38)	Responder hbA1c change (n = 38)	p
HbA1c (mmol/mol)	74 (66–84)	101 (86–112)	**<0.001**
HbA1c (%)	8.9 (8.2–9.8)	11.4 (10.0–12.4)	**<0.001**
Fasting glucose (nmol/l)	10.6 (8.9–12.4)	14.0 (11.4–16.3)	**<0.001**
% male	40%	50%	0.35
Age of diagnosis (years)	50 (38–55)	47 (40–51)	0.12
Duration diabetes (years)	5 (2–7.25)	7(4–12)	**0.04**
BMI (kg/m^2^)	41 (35–46)	40 (35–43)	0.30
Creatinine (µmol/L)	74 (60–91)	67 (55–86)	0.23
Triglycerides (mmol/L)	2.3 (1.7–3.1)	2.0 (1.5–2.6)	0.36
Fasting C-peptide (nmol/L)	1.85 (1.55–2.36)	1.28 (1.17–2.14)	**0.002**

Median (IQR) or %.

### Apparent predictors of response will differ depending on response definition chosen

**Figure 2:**
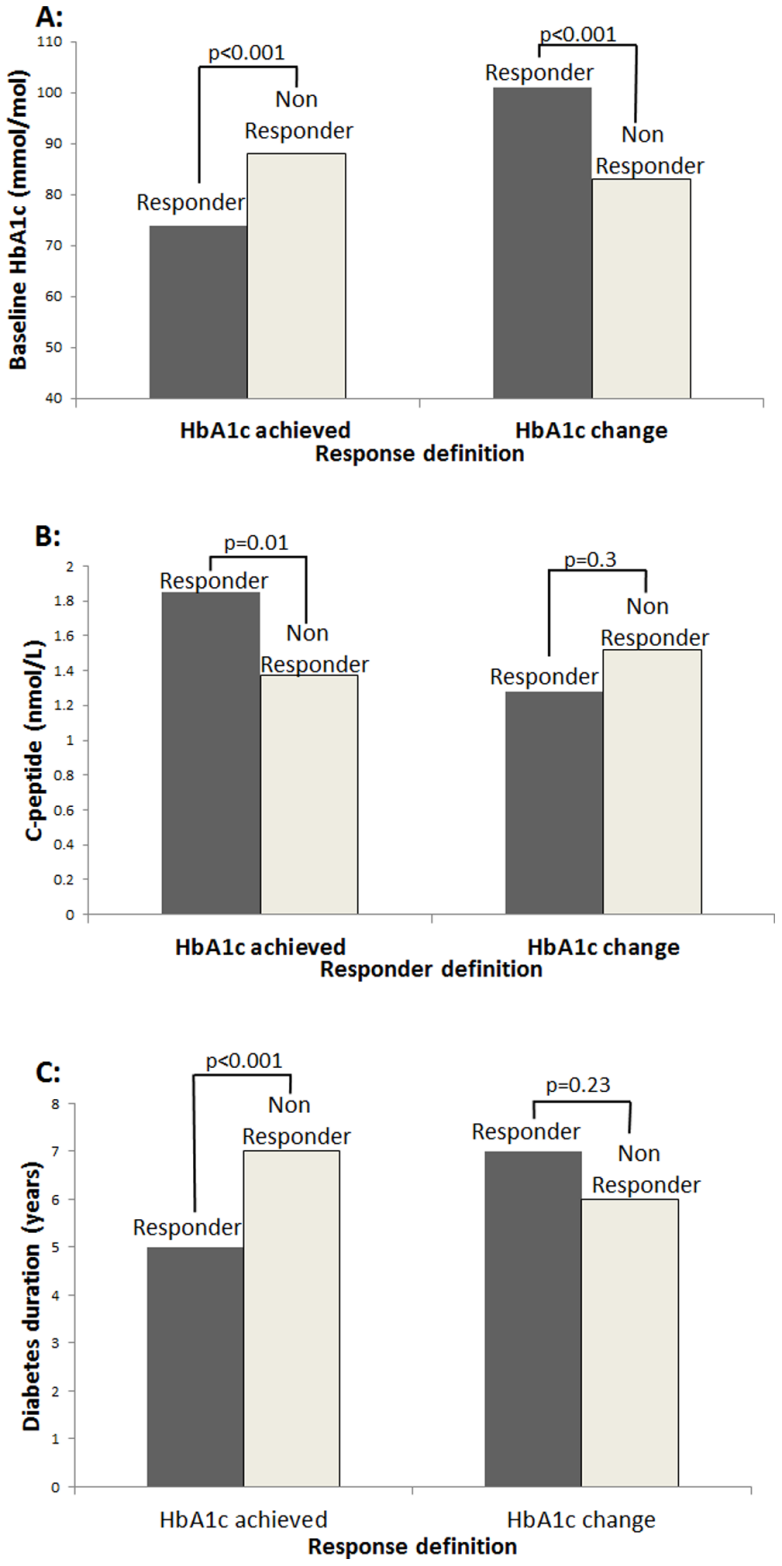
Comparison of baseline HbA1c (a), diabetes duration (b) and C-peptide (c) in ‘responders’ and ‘non-responders’ to GLP-1A defined by HbA1c achieved or HbA1c change. Responders n = 38, non responders n = 131.

Where treatment response is based on HbA1c achieved, low HbA1c and fasting glucose, high C-peptide and shorter duration of diabetes are associated with good glycaemic response to therapy. However when using HbA1c change to define response, the trend for these variables is in the opposite direction (higher HbA1c and glucose, lower C-peptide and longer diabetes duration in responders) (**Figure 2** **,** glucose not shown). Other baseline variables are not associated with response group using either definition (p>0.09 for all, **Table S1**).

### A novel combination of HbA1c change and HbA1c achieved may be used to define response groups which do not differ by baseline HbA1c

**Figure 3:**
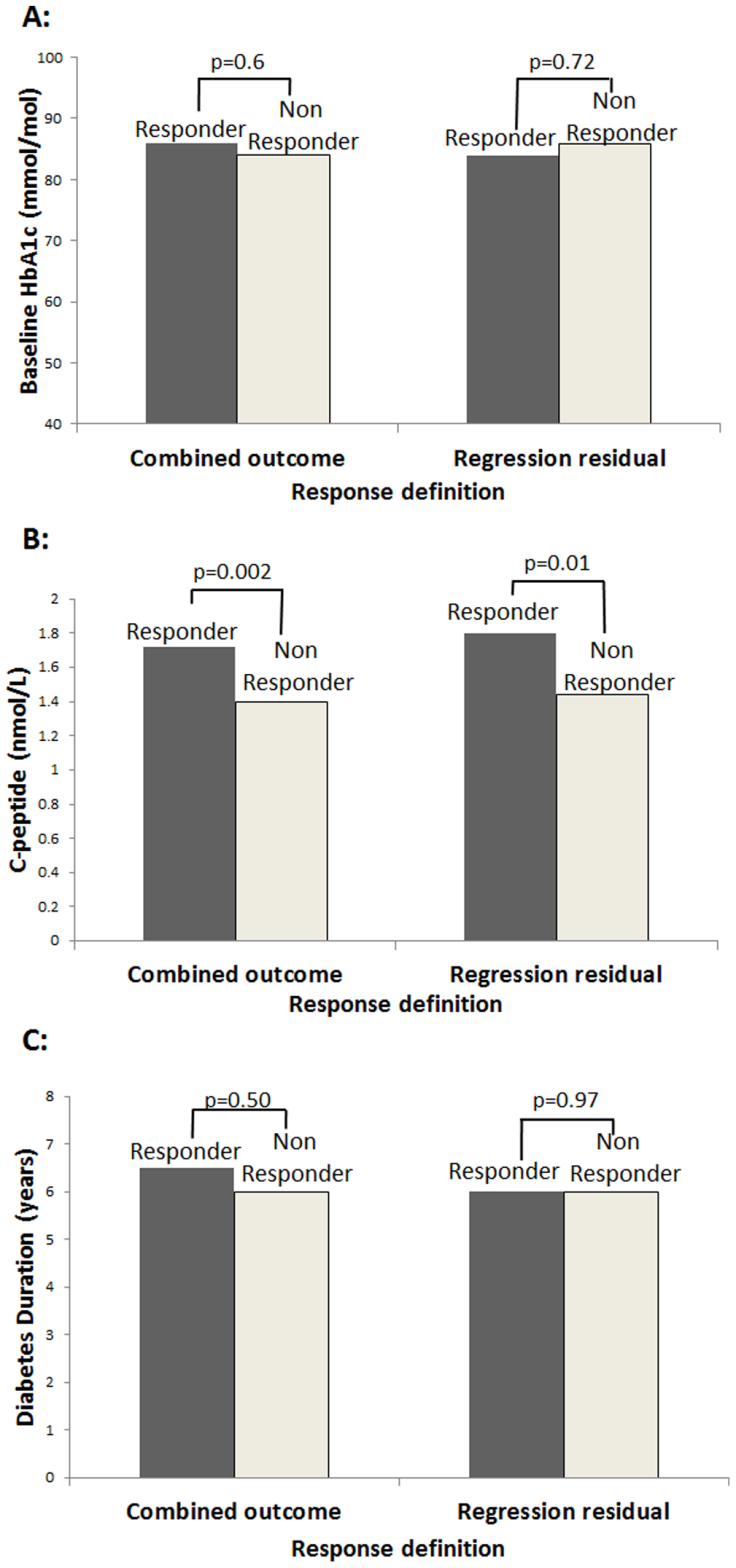
Comparison of baseline HbA1c (a), diabetes duration (b) and C-peptide (c) in ‘responders’ and ‘non responders’ to GLP-1A defined by combined outcome or baseline adjusted HbA1c change (regression residuals). Responders n = 38, non responders n = 131.

By combining both conventional outcomes into a single definition, response groups can be created which do not differ by baseline (see methods). The best 38 responders using this method have similar baseline HbA1c to non-responders (median 86.0 vs 84.0 mmol/mol (10.0 vs 9.8%) responders/non responders, p = 0.60, **Figure 3**). This is consistent across a range of responses; for example when comparing the best 10%, 30% and 60% responders defined using this method with the corresponding ‘non responders’ (remaining participants), baseline HbA1cs do not differ (≤2 mmol/mol (0.2%) difference in median HbA1c and p>0.63 for all). Responders defined this way differ from ‘non-responders’ only by C-peptide (**Figure 3**) (median C-peptide 1.72 vs 1.40 nmol/L responders vs non responders, p = 0.002, p for other baseline variables >0.1 for all).

### This combined outcome method identifies similar individuals and associated characteristics to response groups defined by baseline adjusted HbA1c change

Defining response using linear regression baseline adjusted HbA1c change identifies predominantly the same individuals as the above ‘combined outcome’ method (74% responder agreement, kappa statistic 0.66 (‘substantial agreement’), **Figure S1**). Characteristics of responders and non responders defined using this method are also similar (**Figure 3**): median baseline HbA1c 84.0 vs 85.9 mmol/mol (9.8 vs 10.0%) responders vs non responders, p = 0.72, median C-peptide 1.80 vs 1.44 nmol/L, p = 0.01, p>0.3 for other baseline characteristics). Agreement between regression residuals and the combined method is high across a range of response (best 10% responders agreement 73%, best 50% responders agreement 88%).

## Discussion

This study demonstrates that defining responders to glucose lowering therapy by HbA1c change or HbA1c achieved will identify different individuals, with different baseline glycaemia and associated characteristics. We suggest a simple practical alternative combining HbA1c change and HbA1c achieved that may be used to identify response groups that are independent of baseline HbA1c.

In our study fewer than 40% of the top quartile of responders by one definition of glycaemic response were also responders by the alternative definition. This means studies categorising response to treatment may compare very different response groups should they choose alternate definitions of glycaemic response. Baseline HbA1c, a major source of potential confounding, is markedly different in responders defined by different definitions. This is consistent with previous research that has shown that baseline HbA1c strongly influences response to glucose lowering therapies, with high baseline associated with greater HbA1c fall but less likelihood of achieving glycaemic targets [3]–[5]. Many studies aiming to identify predictors of response do not adjust for baseline glycaemia; this may contribute to marked variation in the findings of these studies, for example both low and high diabetes duration has been reported to be associated with good response to GLP-1 agonist therapy [6]–[8], while others find no association [9], [10].

Our findings are also important in defining response in clinical care. Almost all participants (89%) with baseline HbA1c ≥90 mmol/mol (10.4%) in this study achieved an HbA1c fall of ≥11 mmol/mol (1%), a criteria for continuing therapy beyond 6 months in the UK [1]), but few (15%) with this baseline HbA1c will achieve an HbA1c under 58 mmol/mol (7.5%), the target for glycaemic control set by the same organisation. The converse is true for those with low baseline glycaemia.

Failure to compare patients with similar baseline HbA1c in studies of treatment stratification may lead to the finding of associations between a variable and treatment response that are in fact simply due to an association between the ‘predictive variable’ and baseline glycaemia. This will result in opposite directions of effect if response is defined by target HbA1c achieved rather than absolute HbA1c change. Where a baseline characteristic is a true predictor of response but has the opposite relationships with baseline HbA1c the association with treatment response may be missed due to negative confounding. This is seen with C-peptide in our study, which is more strongly associated with response when comparing participants with similar baseline HbA1c. While it is possible to adjust results for baseline HbA1c when comparing ‘responders’ and ‘non responders’ to glucose lowering therapy, this has the potential to increase error [11]–[13]. When recruiting extremes of response, which will have greater power for physiological studies, it is preferable to compare participants with similar baseline glycaemia.

We have demonstrated a very simple and practical way to define response groups to glucose lowering therapy that do not differ on baseline HbA1c, by combining HbA1c change and HbA1c achieved. This method identifies similar participants and characteristics to using response groups based on HbA1c adjusted response (regression residuals). However unlike regression this method can be used to create clear clinical criteria and a mathematical calculation is not needed to define the responder group for each participant. This is a major advantage for studies recruiting participants into response groups for phenotyping. This method can also be used as a non-parametric method for the examination of existing datasets, which could have potential advantages where regression assumptions are not met.

While this study is limited to one glucose lowering treatment class it is likely our findings will apply to all other glucose lowering medicines; baseline HbA1c is associated with response to all glucose lowering interventions including placebo [3], [4], [14]. This method could also be applied to treatment stratification in other conditions where baseline may influence apparent treatment response, such as the treatment of hyperlipidaemia or hypertension [15], [16]. For reasons of simplicity we have defined ‘non responders’ to therapy in this article as those not meeting criteria for the responder group. However equal comparison groups with poor response can be defined by again specifying a combined threshold, for example failure to achieve the 60^th^ centile of HbA1c change (−16 mmol/mol) and HbA1c achieved (69 mmol/mol) will define a comparison group of 38 non responders in this cohort.

A potential disadvantage of this method is that in studies including a wide range of baseline HbA1c it may preferentially select those with average baseline HbA1c as responders: a participant with a very high baseline will be unlikely to achieve a low on treatment HbA1c, a participant with a low baseline may be unlikely to have a large HbA1c fall. There may be advantages to restricting phenotyping to individuals with similar baseline glycaemia when studying physiology.

A potential limitation of this study is that differences in baseline HbA1c observed between different response groups in this study may have been exaggerated by the study design. We have excluded participants with other treatment change from this analysis as it is not possible to know what the response to GLP-1 therapy would have been had other therapy remained stable. Those with low baseline glycaemia and good response may have been preferentially excluded as they will be more likely to have hypoglycaemia and stop an adjacent therapy than those with high baseline. However this appears to have little effect: including participants who have reduced concurrent therapies does not substantially change the association between baseline HbA1c and glycaemic response (**Table S2**). Repeating our analysis including those who discontinued other therapy does not substantially change our results. Although this study suggests C-peptide but not other examined participant characteristics are associated with glycaemia response to GLP-1 therapy these results are based on a small cohort examining extremes of response over a short (3 month) timescale to address a methodological question, further research will be needed to determine if these associations are of relevance to clinical practice.

In summary our study demonstrates that the outcome of studies aiming to identify predictors of glycaemic response to glucose lowering therapy may depend on how treatment response is defined. Studies defining groups of responders or non responders to glucose lowering therapy should use alternative definitions of response which minimise the influence of baseline HbA1c.

## Supporting Information

Figure S1 **HbA1c achieved on therapy against HbA1c change from baseline, demonstrating difference in responders identified by baseline adjusted HBA1c change (linear regression) and by the combined method.**
(PPTX)

Table S1 **A:** Comparison of ‘responders’ and ‘non responders’ to GLP-1A defined by HbA1c achieved **B:** Comparison of ‘responders’ and ‘non responders’ to GLP-1A defined by HbA1c change
(DOCX)

Table S2 **The effect of excluding participants with co-treatment reductions on baseline HbA1c:HbA1c change association (linear regression HbA1c change on baseline hbA1c).**
(DOCX)
