# Supplementary material for: Identifying Good Responders to Glucose Lowering Therapy in Type 2 Diabetes: Implications for Stratified Medicine
Source: PLoS One. 2014 Oct 23;9(10):e111235. doi: 10.1371/journal.pone.0111235 (PMC4207765; doi:10.1371/journal.pone.0111235)
Supplement: Figure S1 — HbA1c achieved on therapy against HbA1c change from baseline, demonstrating difference in responders identified by baseline adjusted HBA1c change (linear regression) and by the combined method. (PPTX) [file pone.0111235.s001.pptx]

## Slide 1
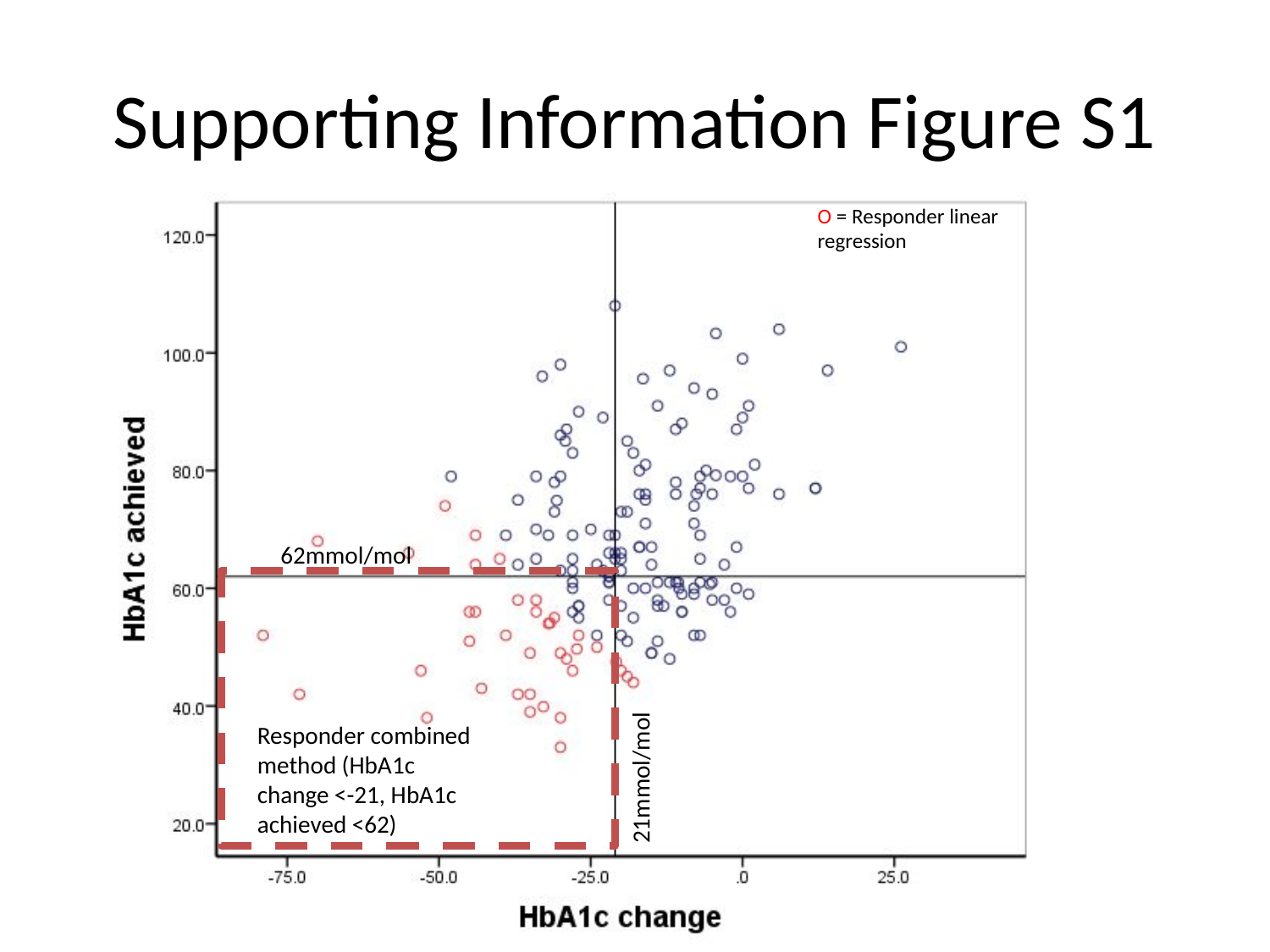

# Supporting Information Figure S1
O = Responder linear regression
62mmol/mol
Responder combined method (HbA1c change <-21, HbA1c achieved <62)
21mmol/mol
