# Supplementary material for: Identifying Good Responders to Glucose Lowering Therapy in Type 2 Diabetes: Implications for Stratified Medicine
Source: PLoS One. 2014 Oct 23;9(10):e111235. doi: 10.1371/journal.pone.0111235 (PMC4207765; doi:10.1371/journal.pone.0111235)
Supplement: Table S1 — A: Comparison of ‘responders’ and ‘non responders’ to GLP-1A defined by HbA1c achieved B: Comparison of ‘responders’ and ‘non responders’ to GLP-1A defined by HbA1c change (DOCX) [file pone.0111235.s002.docx]

**Table S1**

**Table S1A:** Comparison of ‘responders’ and ‘non responders’ to GLP-1A defined by HbA1c achieved

|  | Responder HbA1c achieved (n=38) | Non responder HbA1c achieved (n=131) | P value |
| --- | --- | --- | --- |
| HbA1c baseline (mmol/mol) | 74 (66-84) | 88 (78-101) | **<0.001** |
| HbA1c baseline (%) | 8.9 (8.2-9.8) | 10.2 (9.3-11.4) | **<0.001** |
| Fasting glucose | 10.6 (8.9-12.4) | 12.5 (10.3-15.3) | **0.003** |
| % male | 40% | 55% | 0.12 |
| Age of diagnosis | 50 (38-55) | 45 (40-51) | 0.085 |
| Duration diabetes | 5 (2-7.25) | 7 (4-10) | **0.011** |
| BMI | 41 (35-46) | 39 (35-44) | 0.22 |
| Creatinine | 74 (60-91) | 70 (56-83) | 0.19 |
| Triglycerides | 2.3 (1.7-3.1) | 2.1 (1.5-2.9) | 0.44 |
| Fasting C-peptide | 1.85 (1.54 – 2.55) | 1.37 (1.18 – 1.80) | **<0.001** |

**Table S1B:** Comparison of ‘responders’ and ‘non responders’ to GLP-1A defined by HbA1c change

|  | Responder HbA1c change (n=38) | Non responder HbA1c change (n=131) | P value |
| --- | --- | --- | --- |
| HbA1c baseline (mmol/mol) | 101 (86-112) | 83 (71-91) | **<0.001** |
| HbA1c baseline (%) | 11.8 (10.0-12.4) | 9.7 (8.6-10.5) | **<0.001** |
| Fasting glucose | 14.0 (11.4-16.3) | 11.7 (9.7-13.8) | **0.004** |
| % Male | 50% | 52% | 0.95 |
| Age of diagnosis | 47 (40-51) | 46 (40-52) | 0.67 |
| Duration diabetes | 7(4-12) | 6 (3-9) | 0.30 |
| BMI | 40 (35-43) | 40 (35.5-44.8) | 0.57 |
| Creatinine | 67 (55-86) | 71 (58-84) | 0.54 |
| Triglycerides | 2.0 (1.5-2.6) | 2.2 (1.5-3.1) | 0.51 |
| Fasting C-peptide | 1.28 (1.17-2.14) | 1.52 (1.23-1.89) | 0.23 |
