# Supplementary material for: Identifying Good Responders to Glucose Lowering Therapy in Type 2 Diabetes: Implications for Stratified Medicine
Source: PLoS One. 2014 Oct 23;9(10):e111235. doi: 10.1371/journal.pone.0111235 (PMC4207765; doi:10.1371/journal.pone.0111235)
Supplement: Table S2 — The effect of excluding participants with co-treatment reductions on baseline HbA1c:HbA1c change association (linear regression HbA1c change on baseline hbA1c). (DOCX) [file pone.0111235.s003.docx]

**Table S2**: The effect of excluding participants with co-treatment reductions on baseline HbA1c:HbA1c change association (linear regression HbA1c change on baseline hbA1c).

|  | Regression B | Pearson r^2^ |
| --- | --- | --- |
| Restricted cohort (no treatment change) | -0.5 | 0.32 |
| Restricted cohort + any reduction in glucose lowering co-therapy | -0.49 | 0.27 |
